# Prediction of Tibial Rotation Pathologies Using Particle Swarm Optimization and K-Means Algorithms

**DOI:** 10.3390/jcm7040065

**Published:** 2018-03-28

**Authors:** Murat Sari, Can Tuna, Serkan Akogul

**Affiliations:** 1 Department of Mathematics, Yildiz Technical University, Istanbul 34220, Turkey; cantuna22@gmail.com; 2 Department of Statistics, Yildiz Technical University, Istanbul 34220, Turkey; sakogul@yildiz.edu.tr

**Keywords:** tibial rotation pathology, K-means clustering, particle swarm optimization

## Abstract

The aim of this article is to investigate pathological subjects from a population through different physical factors. To achieve this, particle swarm optimization (PSO) and K-means (KM) clustering algorithms have been combined (PSO-KM). Datasets provided by the literature were divided into three clusters based on age and weight parameters and each one of right tibial external rotation (RTER), right tibial internal rotation (RTIR), left tibial external rotation (LTER), and left tibial internal rotation (LTIR) values were divided into three types as Type 1, Type 2 and Type 3 (Type 2 is non-pathological (normal) and the other two types are pathological (abnormal)), respectively. The rotation values of every subject in any cluster were noted. Then the algorithm was run and the produced values were also considered. The values of the produced algorithm, the PSO-KM, have been compared with the real values. The hybrid PSO-KM algorithm has been very successful on the optimal clustering of the tibial rotation types through the physical criteria. In this investigation, Type 2 (pathological subjects) is of especially high predictability and the PSO-KM algorithm has been very successful as an operation system for clustering and optimizing the tibial motion data assessments. These research findings are expected to be very useful for health providers, such as physiotherapists, orthopedists, and so on, in which this consequence may help clinicians to appropriately designing proper treatment schedules for patients.

## 1. Introduction

Scientific problems encountered in nature are generally modeled mathematically. Thus, to deal with these problems, production of related algorithms has been of great attraction since the advent of computers. This is the case in biomechanical problems as well. The knee motion has great importance and various approaches have been utilized to define the range of motion of it [1]. Since the literature reports that there exists a powerful link between the tibial rotation and knee injuries [2,3], it is important to predict tibial rotation types of pathologies during daily examination.

As pointed out by Dye [4] the knee joint is among the most substantial joints in the musculoskeletal system. To quantify the knee joint, instrumented arthrometry is a commonly used technique. Special attention has been paid to knee joint laxity and several methods have been used to define the range of motion of the knee joint especially flexion and extension [1,5,6,7,8,9,10,11].

Although there exist various studies in the literature, few of them have analyzed the tibial motions involving the internal and external rotations [3,8,12,13,14,15,16,17]. However, it was concluded that specific knee injuries are led by various forms of internal and external tibial rotations. Owing to external rotation related to knee extension, high internal rotation during the stance phase of walking may postpone the external rotation while the knee stretches. This has the ability to increase torsional joint stresses through the tibial shaft and, in turn, lead to knee injury rotation [2,18].

Examination of the tibial motion is usually difficult for clinical points of view. Even though there exist stunning investigations in the literature, the pathological interval of the tibial rotations has not been evidently examined yet. In the literature, there have been several methods [6,16,17,19,20,21,22,23,24,25,26,27,28,29,30] to analyze the tibial motion. In addition to their notable advantages, the majority of them have many drawbacks such as being expensive, difficult to use, time consuming, and having constraints in daily use etc. In this instance, optimization modeling can be borne in mind in addition to those methods. There are some methods at the optimization stage, particularly particle swarm optimization (PSO). The PSO modeling which is being used very often in recent years makes an optional approach for many data processing treatment. This paper combines the PSO and KM clustering algorithms (PSO-KM) in predicting the tibial rotation pathologies type. To the best knowledge of the authors, the idea of the PSO-KM has not been applied to forecast the tibial rotation pathologies through the physical parameters so far. Since the PSO-KM algorithm is more flexible and does not need expertise on statistics, it has been proposed for the reliable data treatment and following interpretations in the present article. The PSO-KM, as general optimization clustering algorithm, makes the estimation process possible for various patterns through the available data of a problem area by predicting the tibial rotation pathologies among the data patterns. The main purpose of this article is to investigate pathological subjects from a population. To accomplish this a hybrid algorithm consisting of a combination of two essential algorithms, PSO and KM clustering algorithms has been produced.

## 2. Materials and Methods

### 2.1. Subjects and Study Design

**Figure 1:**
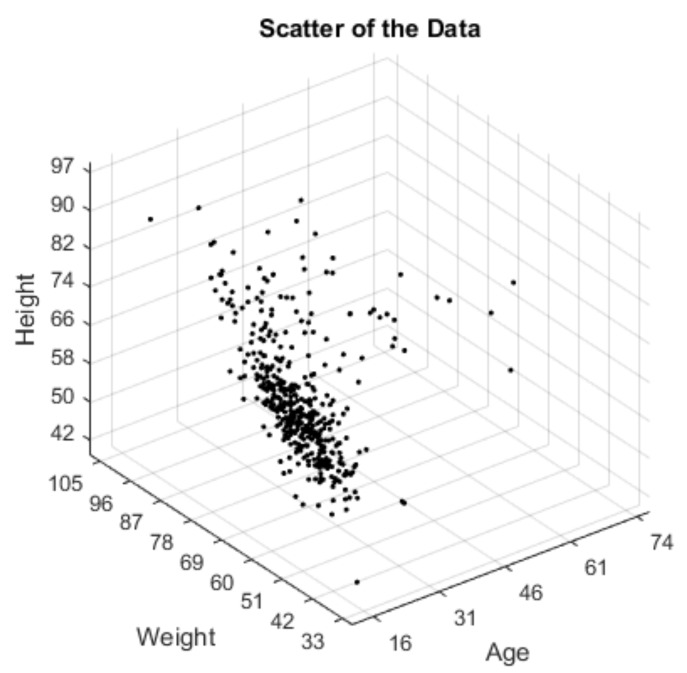
Scatter plot of the data of the subjects with age, weight and height parameters.

The data for 484 healthy subjects were provided from the literature [16,30,31]. The measurement method of the dataset was explained in the work of Cimbiz et al. [30]. The subjects do not have any health problems or knee joint injuries. The data includes measurement of age, weight and height information of 484 volunteers. The age, weight and height values of each subject are shown in Figure 1.

**Figure 2:**
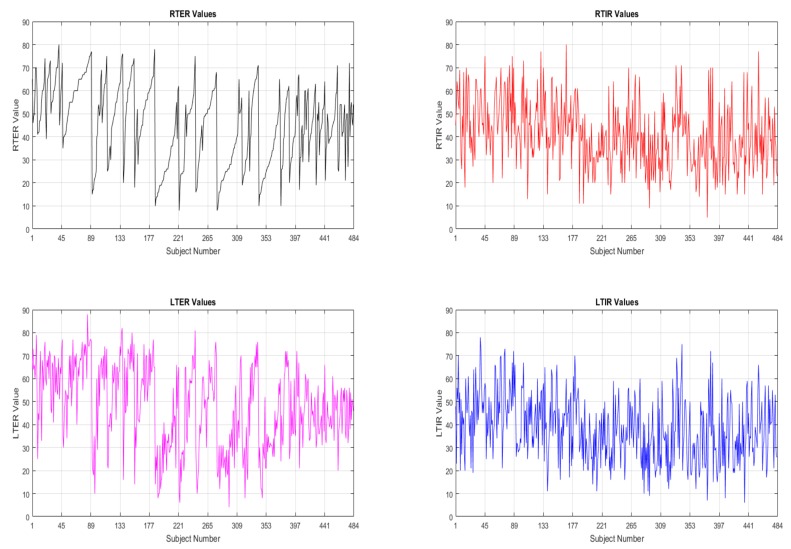
Tibial rotation values of every subject.

In the data tibial rotation values of each subject were given as right tibial external rotation (RTER), right tibial internal rotation (RTIR), left tibial external rotation (LTER), left tibial internal rotation (LTIR) as seen in Figure 2. Totally, for every subject, it was 7 parameters as 3 of them are physical factors (age, weight and height) and 4 of them are tibial rotation values (RTER, RTIR, LTER and LTIR). The physical parameters are input and the rotation values are output.

The pragmatic aim of the paper is to discover the tibial rotation pathologies from a population by using the PSO-KM clustering algorithm, different physical characteristics. Primarily, 3 physical factors age, weight and height have been examined for the RTER values. Then, the same analysis has been carried out for the other variables RTIR, LTER, LTIR.

Since clustering is determined as the basic principle, the subjects are divided into three clusters by age and weight parameters. Subjects that their ages are greater than 30 are identified as the first cluster. The remaining subjects that their ages are less than 30 are divided into two clusters by the weight parameter. Subjects that their weights are less than 60 kg (subjects which have 1.70 height) are identified as the second cluster. Again, the remaining subjects that their weights are greater than 60 kg are identified as the third and the last cluster in Table 1.

**Figure 3:**
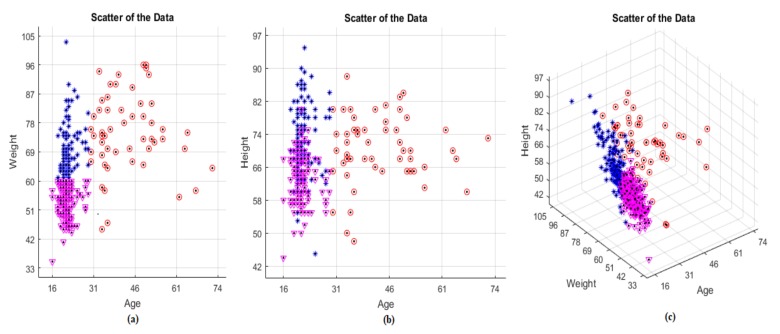
Clusters of data; (**a**) Age-Height view; (**b**) Age-Weight view; (**c**) View of all data.

Since clustering is done according to age and weight parameters, showing all data in age-weight graph will be more imaginable. In Figure 3, red, purple and blue parts represent the Cluster 1, Cluster 2 and Cluster 3, respectively.

Clustering has been done according to the scatter of the data. For example, the right side, which represents the subjects are older than 30 years, seems to be more dispersed than the left side. So, this part has been accepted to be Cluster 1.

In the meantime, the left side could be thought of as another cluster but two clusters were very easy for this problem. To have three clusters, the left side is divided into two by considering the weight parameter. The effect of height and weight parameters on clustering is almost the same, as seen in Figure 3. Since Figure 3 is displayed in this way, the left side of age-weight graph is divided into two clusters. The purple part which is below the left side represents the subjects are of less than 60 kg of weight while the blue part represents the subjects which are of greater than 60 kg of weight. Thus, establishment of the clustering is seen in Table 1.

Once the clusters have been identified, the number of people for each type is counted in each cluster. This calculation has been made individually for each rotation type RTER, RTIR, LTER and LTIR. Each rotation has been divided into 3 regions according to whether it is pathological or not.

When the angle of the tibial rotation remains between 0 and 20 degrees, the corresponding subject is pathological in which each rotation type is accepted to be Type 1. If the angle of the tibial rotation of the adult subjects is between approximately 20 and 65 degrees, the subject who has angle in this interval is known to be normal thus, this type is called Type 2. Likewise, the case over approximately 65 degrees is abnormal and thus pathological. This part is accepted to be Type 3 again for each rotation type. The ranges of all types and the number of subjects in each rotation type are shown in Table 2. After all these calculations have been done, the number of types in each cluster has been examined as seen in Table 3. All classifications of the subjects done in above are based on the criterion of being pathological or non-pathological [32,33,34].

As seen from the results of Table 3, the algorithm to be used in this study aims to find the correct number of subjects in the clusters and find out the pathological and non-pathological values (Type 1, Type 2 and Type 3) in those clusters.

Since clusters are categorized according to the physical factors, when the rotation measurements are categorized according to the clusters, it will be examined whether there is a relation between these pathological and the non-pathological cases physical factors. Table 2 informs that these anomalies are RTER-Type 1, RTER-Type 3, RTIR-Type 1, RTIR-Type 3, LTER-Type 1, LTER-Type 3, LTIR-Type 1 and LTIR-Type 3.

By applying the PSO-KM algorithm to this data, both accuracy of the clustering of the data and effects of the physical information on the tibial motion are investigated. In the literature, various versions of the PSO-KM algorithm were produced for various problems in different areas of science. Separately or combined, PSO and KM clustering algorithm are already reported to be very successful algorithms for their own problems [35,36,37,38,39,40,41,42,43,44,45,46,47,48,49,50]. A combined version of this algorithm and its computer codes have been successfully produced in this work. One of the greatest contributions of this study is that the algorithm is applied to the tibial rotation data for the first time.

### 2.2. K-Means Algorithm

**Figure 4:**
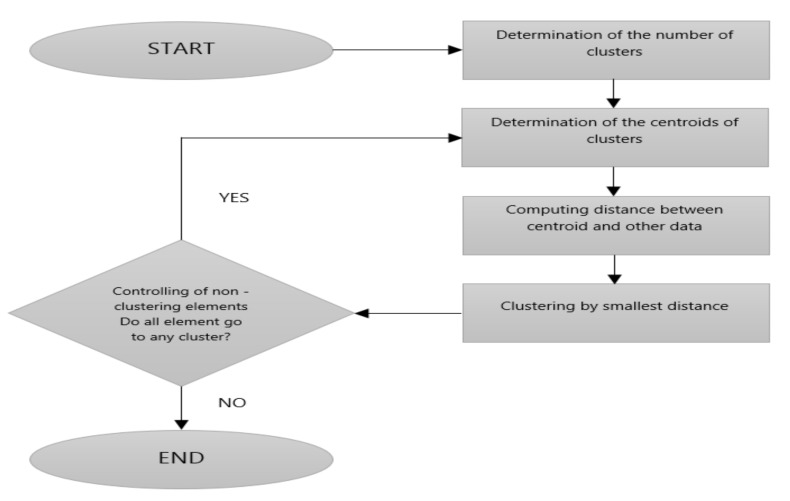
Flow diagram of the K-means (KM) algorithm.

The KM algorithm was first explored by MacQueen [51]. The algorithm is one of the fastest, simplest and commonest in the literature for clustering problems [52,53,54,55]. The clustering algorithm divides N sample data into K clusters by controlling the distance between each other. Since the points that the closest to each other are clustering-based algorithm, the KM must have an objective function and this objective function will be minimization problem. More details on the algorithm can be found in the literature [51,52,53,54,55]. The flow diagram of the KM clustering algorithm is given in Figure 4.

### 2.3. Particle Swarm Optimization

**Figure 5:**
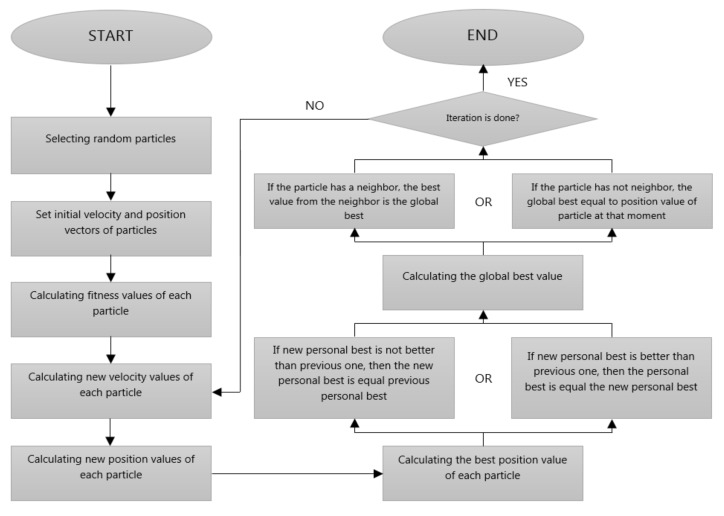
Flow diagram of particle swarm optimization (PSO).

Particle swarm optimization (PSO) is a population-based, evolutionary optimization algorithm found by Kennedy and Eberhart [56]. They inspired from the collective movement of birds and fishes. These animals have a major role in the development of the algorithm to escape from dangerous situations or to search food by looking at each other. The PSO is a very fast and more successful method than other optimization algorithms because it requires relatively fewer parameters and is less likely to find local minimum points as a solution [57,58]. In general, how the PSO works is shown in the flow diagram in Figure 5.

The main principle of the PSO is that these elements try to find the optimum solution by selecting random elements from the given space. In the PSO, these elements that selected randomly are called “particle”. These randomly selected particles search solution space using information of their neighborhood, personal information, and randomness. Because of these 3-components, the particles go elsewhere at the end of each iteration. This 3-component formula, which takes the particle at any point at the end of each iteration, is considered to be the velocity vector of the particle. The position where the particle goes is the position vector of the particle. These velocity and position vectors are initialized as the information of initial values of the selected particle [59,60,61,62,63]. Because of each iteration, the position and velocity vectors are updated as follows:

(1) $$V_{i}^{t+1}=wV_{i}^{t}+c_{1}r_{1}\left(P_{best}-x_{i}\right)+c_{2}r_{2}\left(G_{best}-x_{i}\right)$$

(2) $$X_{i}^{t+1}=X_{i}^{t}+V_{i}^{t+1}$$

t: Iteration number;
*w*: Inertia factor;
$c_{1}$: Cognitive parameter;
$c_{2}$: Social parameter;
$r_{1}$: Random numbers between (0, 1);
$r_{2}$: Random numbers between (0, 1);
$P_{best}$: The best local value of each particle;
$G_{best}$: The best value of swarm

The particles hold their best value in their memories. This value is called as $P_{best}$. These values are calculated by the fitness function. When the problem is minimization, the smallest value is $P_{best}$. If the problem is maximization, then the biggest value is $P_{best}.$ $P_{best}$ of each particle is listed and the fittest value according to the fitness function is selected as $G_{best}$ [57,58,59,60,61,62,63].

In the experimental studies [64], the most appropriate value of w was accepted as approximately 0.73. Again, it may change depending on the problem type but t is the most suitable value, usually 20−30. In the same way, in the experimental studies, $c_{1},c_{2}$ values are bounded as $c_{1}+c_{2}=4$ and $c_{1}$ and $c_{2}$ usually take the same value as $c_{1}=c_{2}=1.49$ [64].

As a result, random particles are selected from solution space. These particles are looking randomly for a solution. Particles move with their best value, neighbor’s best value and randomness. Because of this movement, the new position of each particle is determined. At the end of the stated number of iterations, the best one of the values found in the fitness function is accepted to be a solution in the PSO.

### 2.4. The PSO-KM Algorithm

Optimization is mostly used in biomechanical problems to analyze system identification problems, predict human motion and so on. Biomechanical optimization problems usually have multiple local minima, making it difficult to find the best solution. Hybridization of the PSO with KM clustering algorithm (PSO-KM) is explained in this section. Even if the hybrid algorithm has been seen to be applied in some scientific problems [35,36,37,38,40,41,47,48,50], it is the first time that the algorithm is applied to the tibial rotation. The KM algorithm is a very successful and iterative algorithm. Likewise, the PSO is also a very successful optimization method. The common feature of these two algorithms is their iterative structure. At the cluster center, finding steps of the KM clustering algorithm can be made better by using the PSO approach. Hereby, better cluster center can be found. Thus, the main idea of their combination has been developed in this way. The literature tells us that different methods were applied to deal with the tibial rotation [16,17,30]. However, the currently proposed PSO-KM algorithm has been utilized for the first time in this area. 

The PSO-KM algorithm provides a more realistic approach to human nature and recognition than conventional methods. The current algorithm can also be used successfully in very large fields of science such as image recognition, signal processing, financial and economical modeling and control systems. Possibility and ease of the use in many fields make the present optimization approach an ideal solution for many applications.

As declared in the next section, there is considerable relation existing between some of the physical factors even the relation is relatively very strong for all the design variables. The data consists of age, weight and height values as well as left and right external and internal tibial rotations taken from 484 healthy subjects. At the beginning, the three parameters alone have been used to explore the rotation types, i.e., either pathological or non-pathological. The general equation of the PSO becomes:

(3) $$V_{ij}^{t+1}=wV_{ij}^{t}+c_{1}r_{1}\left(P_{best}-x_{ij}\right)+c_{2}r_{2}\left(G_{best}-x_{ij}\right)$$

(4) $$X_{ij}^{t+1}=X_{ij}^{t}+V_{ij}^{t+1}$$

where j represents the number of dimensions. As an example, $V_{62}^{4}$ represents the velocity vector of 6th particle in 2nd dimension at 4th iteration. If data is to be spoken, our data has 3 dimensions. It will be age, body mass and height are the first, the second and the third input (or independent) variables, respectively. So, $V_{62}^{4}$ represents the velocity vector of the sixth one from selected particles for solution on weight parameter in the 4th dimension. In the KM clustering algorithm, our data have the number of clusters K=3 and the number of subjects N=484. Once the problem is designed in this way, the KM clustering algorithm distinguishes these parameters from each other by the mentality of being similar. Each one of the rotation types RTER, RTIR, LTER, and LTIR is sorted by just age parameter, respectively. Because the clustering was done according to the parameter age. The results of the KM clustering and values of each rotation (as Type 1, Type 2 and Type 3) were compared.

## 3. Results

**Figure 6:**
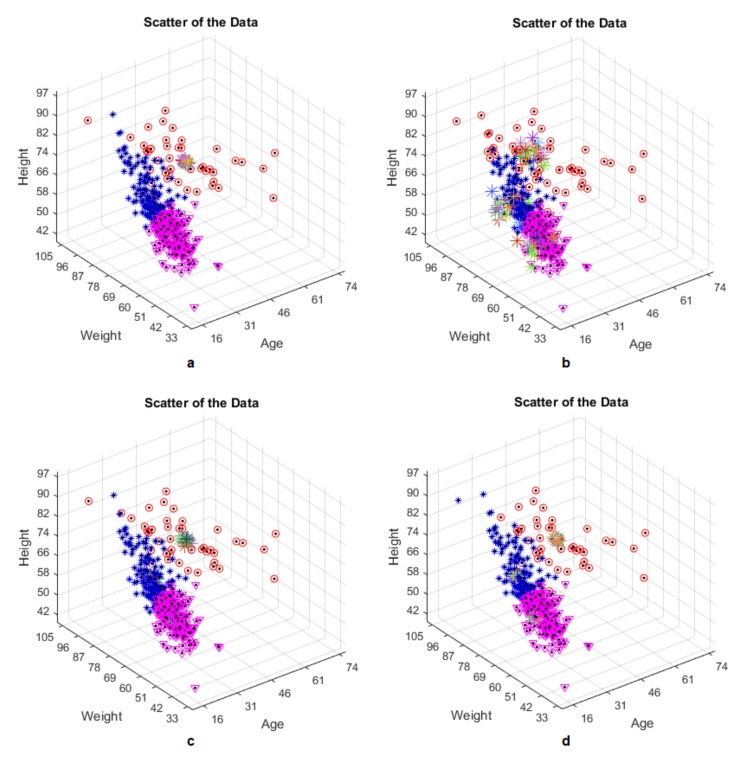
One of the cluster results for: (**a**) right tibial external rotation (RTER); (**b**) right tibial internal rotation (RTIR); (**c**) left tibial external rotation (LTER); (**d**) left tibial internal rotation (LTIR) values.

The PSO-KM algorithm produced is designed to cluster the rotations RTER, RTIR, LTER and LTIR. The rotation values in Table 4 are the actual values of the clustering, taking RTER, RTIR, LTER and LTIR values into consideration. The algorithm will try to find the physical properties of the individual clustered data for each rotation value. The algorithm finds out whether the data for each rotation value are clustered correctly. For each type of the rotations RTER, RTIR, LTER, LTIR, clustering results have been presented in Figure 6, respectively.

**Figure 7:**
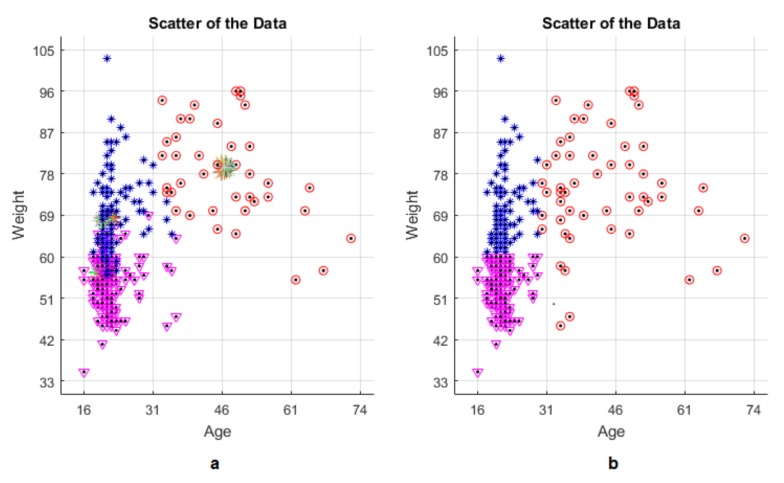
(**a**) Comparison of the computed results and (**b**) the real values for LTIR.

It is good idea to observe scattering behavior of the data both realistically and computationally. The computed results and the real values have been presented in a comparative way in Figure 7, respectively. It is realized that the calculated results of all clusters agree well with the real results. As shown, the algorithm seems to have very small errors for the red and purple points. The red and purple points represent the Cluster 1 and Cluster 2. In the blue points (Cluster 3), there is a relatively larger error than the ones in the other two cases. This is because the data is very close to each other and the centroids of the clusters. As can be seen from this comment, it can be predicted that the produced algorithm gives better results for both Cluster 1 and Cluster 2 than the Cluster 3.

As seen in Table 4, the produced results of the PSO-KM algorithm are generally good agreement with the results of the KM algorithm. For example, when the LTIR values are considered, the number of people in Cluster 1 is 52 while the results produced by the PSO-KM and the KM algorithms are 46 and 45 people, respectively. For Cluster 1, the success rates of the PSO-KM and the KM algorithms are 88.46% and 86.54%. Similarly, in the same order, the algorithms found the Cluster 2 values consisting of 249 people as 239 and 218 people. The success rates of the algorithms are found to be 95.98% and 88.76% for the Cluster 2, respectively. In the same way, the two algorithms have produced results for the Cluster 3 as 199 and 218 people while the real value is 183 people. The success rates of the algorithms are 91.96% and 83.94% for the Cluster 3. Consideration of the values in Table 4 leads one to make similar discussions for other rotation variables LTER, RTER and RTIR.

In Table 5, the clustering success of the rotation types in the cluster has been taken into consideration. For the LTIR in Cluster 1 consisting of 52 subjects; real values for Type 1, Type 2 and Type 3 are 3, 49 and 0 respectively. Thus, the real ratios of Type 1 and Type 2 are 5.77% and 94.23%, respectively. The PSO-KM algorithm has discovered 42 subjects from 46 ones to be Type 2 whilst both Type 1 and Type 3 are of 2 subjects. The algorithm has success rates for Type 1, Type 2 and Type 3 to be 4.35%, 91.30% and 4.35%, respectively. As revealed from the table, the KM algorithm is seen to produce a similar success rates for the three types. Under the consideration of the results in Table 5; similar discussions can be carried out for other rotation variables LTER, RTER, RTIR in the other two clusters; Cluster 2 and Cluster 3.

With the help of Table 5, a general comparison of the results has been carried out in Table 6. In Table 5, for the LTIR, the ratio for Type 2 in Cluster 1 is 94.23% while the PSO-KM algorithm finds it to be 91.30%. Thus, the PSO-KM algorithm produced a result with 96.89% accuracy as seen in Table 6. In a similar manner, the ratio for Type 2 in Cluster 2 is 87.55% while the algorithm produces it to be 91.22%. Accuracy of the current algorithm is thus found to be 95.98%. Similarly, the ratio for Type 2 in Cluster 3 is 80.33% while the algorithm finds it to be 77.39%, and therefore accuracy of the present algorithm is seen to be 96.34%. As revealed from the table, the KM algorithm seems to produce similar level of accuracies for all cases.

When we consider the results in Table 6, in each cluster, the reason why the performance ratio of Type 2 is high is that there are many subjects in the clusters. So, even if there are little errors in the algorithm, the ratio does not decrease too much. Because of the same reasons, the success of both Type 1 and Type 3 is not as high as previous ones. Because the number of subjects is relatively very few and any little error made will cause the ratio to decrease too much. For example, from Table 4, Type 1 value of the Cluster 1 for the RTIR is 1 person, in the same way, at the results of the PSO-KM algorithm; the Type 1 value of the Cluster 1 for the RTIR is 2 people. Despite the difference of only 1 person, the rate increases from 1.92% to 4.35% (Table 5). Accuracy is of 44.14% could be achieved (Table 6). 

While comparing the PSO-KM algorithm and the KM clustering algorithm, it might be more sensible to get Type 2 instead of both Type 1 and Type 3. Because Type 1 and Type 3 have fewer numbers of elements than Type 2. This leads to large rate changes of the small deviations in the algorithms. The proposed hybrid algorithm, the PSO-KM algorithm, is clearly better than the KM algorithm in the Cluster 2 which has a high number of elements. Cluster 3 has fewer elements than Cluster 2, so the success rate of the PSO-KM is decreased, but its results are still more accurate than the results of the KM algorithm. On the other hand, Cluster 1 has far fewer elements than Cluster 2 and Cluster 3, so the KM produces slightly better results than the PSO-KM in Cluster 1.

In cases where each observation in a dataset has cluster memberships known, external validity measures are used to compare the performance of the clustering algorithms. The external validity measures test the quality of clusters by comparing the results of clustering with the true class labels. The performances of the PSO-KM and the KM algorithms have been measured using the Rand Index [65] encountered as one of the external validity measures. The Rand Index has a value between 0 and 1, and if the Rand Index value approaches 1, indicating an increase in the agreement between the clusters [47]. The Rand Index values produced for the PSO-KM and the KM algorithms have been presented in Table 7. In the table, the cluster validity of the PSO-KM algorithm has been found to be better than the KM algorithm.

## 4. Discussion and Analysis

Since the day that they were found, the PSO algorithm and the KM clustering algorithm have been using as very successful two algorithms in different areas. In this study, combination of the two algorithms has been applied to discover in the tibial rotation pathologies for the first time. It was tested whether it would be successful in this area as is the case other areas of science. Hybridization of the KM clustering with the PSO, so the PSO-KM algorithm, has produced very effective results in the investigation of the tibial rotation pathologies. The application in this field helps clinicians to predict the type of the rotation, that is, pathological or non-pathological. Clustering success was targeted by dividing the rotation values RTER, RTIR, LTER and LTIR into pathological (Type 1 and Type 3) or non-pathological (Type 2) classes. In this problem, the number of clusters for the algorithm is given by the user. Subjects are divided into 3 clusters (Cluster 1, Cluster 2 and Cluster 3) according to age and weight parameters. Using these values, the prediction of the tibial rotation pathologies has been examined through different physical characteristics and the success of the clustering algorithm has been checked. One of the most striking results was that each Type 2 value could be clustered correctly in each cluster for each rotation value. This means that the proposed algorithm works much better when relatively enough data is used. The obtained results are seen to be as expected since the fact that individuals are young, have no disabilities, are the reasons that the subjects increase in Type 2. Since the data is consisting of subjects mostly younger than thirty years old, this work may be relatively less decisive for that subjects who are older than thirty years old in this data.

## 5. Conclusions and Recommendations

This article has investigated the feasibility of the proposed PSO-KM algorithm in predicting the tibial rotation pathologies based on the physical parameters: age, weight, and height. For the first time, this study has predicted the tibial motion pathologies through several physical parameters using the newly combined method. The values of the produced algorithm, the PSO-KM, have been compared with the real values. The hybrid PSO-KM algorithm has been seen to be very successful in optimizing the tibial motion results through the physical criteria. It is concluded that findings are clinically expected to be very beneficial for planning appropriate treatment programs for patients. The current algorithm has been produced for the prediction of the tibial rotation pathologies for the first time. For further research, this study could be divided into more clusters depending on the structure of the data, as the structure of the existing data is limited to have more clusters for a medical point of view. In the future, more clusterable, and thus more informative, results may be found with different datasets.

## Figures and Tables

**Table 1:** Clusters and subject numbers.

	Age	Weight	Height	Number of Subjects
**Cluster 1**	>30	-	-	52
**Cluster 2**	≤30	≤60	≤1.70	249
**Cluster 3**	≤30	>60	>1.70	183

**Table 2:** Type values of each rotation and number of subjects of rotation types.

	RTER	RTIR	LTER	LTIR
Type 1 (≤20°)	39	33	37	51
Type 2 (20°–65°)	391	423	357	414
Type 3 (>65°)	24	28	90	19

**Table 3:** The number of each type in each cluster for every rotation type.

		Cluster 1	Cluster 2	Cluster 3	Total
**RTER**	Type 1	0	17	22	39
	Type 2	50	183	158	391
	Type 3	2	49	3	24
	Total	52	249	183	484
**RTIR**	Type 1	1	7	25	33
	Type 2	48	223	152	423
	Type 3	3	19	6	28
	Total	52	249	183	484
**LTER**	Type 1	1	16	20	37
	Type 2	47	160	150	357
	Type 3	4	73	13	90
	Total	52	249	183	484
**LTIR**	Type 1	3	14	34	51
	Type 2	49	218	147	414
	Type 3	0	17	2	19
	Total	52	249	183	484

**Table 4:** Comparison of the results of the PSO-KM algorithm and the results of the KM algorithm.

		Real Values				PSO-KM Values				KM Values			
		C1	C2	C3	Total	C1	C2	C3	Total	C1	C2	C3	Total
**RTER**	Type 1	0	17	22	39	0	19	27	46	0	7	39	46
	Type 2	50	183	158	391	38	195	158	391	44	206	142	392
	Type 3	2	49	3	54	8	37	2	47	3	36	7	46
	Total	52	249	183	484	46	251	187	484	47	249	188	484
**RTIR**	Type 1	1	7	25	33	2	6	18	26	1	1	22	24
	Type 2	48	223	152	423	40	223	160	423	39	231	154	424
	Type 3	3	19	6	28	4	22	9	35	1	31	4	36
	Total	52	249	183	484	46	251	187	484	41	263	180	484
**LTER**	Type 1	1	16	20	37	1	13	34	48	2	7	33	42
	Type 2	47	160	150	357	35	190	132	357	38	210	110	358
	Type 3	4	73	13	90	2	48	29	79	1	66	17	84
	Total	52	249	183	484	38	251	195	484	41	283	160	484
**LTIR**	Type 1	3	14	34	51	2	10	39	51	3	16	45	64
	Type 2	49	218	147	414	42	218	154	414	42	202	170	414
	Type 3	0	17	2	19	2	11	6	19	0	3	3	6
	Total	52	249	183	484	46	239	199	484	45	221	218	484

C1: Cluster 1; C2: Cluster 2; C3: Cluster 3.

**Table 5:** Comparison of the PSO-KM and the KM algorithms via the success rates.

		Cluster 1			Cluster 2			Cluster 3		
		Real	PSO-KM	KM	Real	PSO-KM	KM	Real	PSO-KM	KM
**RTER**	Type 1	-	-	-	6.82	7.57	2.81	12	14.43	20.7
	Type 2	96.2	82.61	93.6	73.5	77.69	82.7	86.3	84.5	75.5
	Type 3	3.85	17.39	6.38	19.7	14.74	14.5	1.64	1.07	3.72
**RTIR**	Type 1	1.92	4.35	2.44	2.81	2.4	0.38	13.7	9.63	12.2
	Type 2	92.3	86.96	95.1	89.6	88.84	87.8	83.1	85.56	85.6
	Type 3	5.77	8.69	2.44	7.63	8.76	11.8	3.28	4.81	2.22
**LTER**	Type 1	1.92	2.63	4.88	6.43	5.18	2.47	10.9	17.44	20.6
	Type 2	90.4	92.1	92.7	64.3	75.7	74.2	82	67.7	68.8
	Type 3	7.7	5.27	2.44	29.3	19.12	23.3	7.1	14.87	10.6
**LTIR**	Type 1	5.77	4.35	6.67	5.62	4.18	7.24	18.6	19.6	20.6
	Type 2	94.2	91.3	93.3	87.6	91.22	91.4	80.3	77.39	78
	Type 3	-	4.35	-	6.83	4.6	1.36	1.09	3.01	1.38

**Table 6:** Comparison of the PSO-KM and KM algorithms through general accuracy (%).

		Cluster 1		Cluster 2		Cluster 3	
		PSO-KM	KM	PSO-KM	KM	PSO-KM	KM
**RTER**	Type 1	-	-	90.09	41.20	93.30	57.96
	Type 2	85.92	97.37	94.61	88.84	97.87	87.48
	Type 3	22.14	60.34	74.89	73.47	65.24	44.09
**RTIR**	Type 1	44.14	78.69	85.41	13.52	70.50	89.46
	Type 2	94.20	96.84	99.20	98.07	97.08	97.00
	Type 3	66.39	42.29	87.10	64.72	68.19	67.68
**LTER**	Type 1	73.00	39.34	80.56	38.41	62.67	52.98
	Type 2	98.13	97.52	84.89	86.60	82.59	83.87
	Type 3	68.44	31.69	65.23	79.56	47.75	66.85
**LTIR**	Type 1	75.39	86.51	74.38	77.62	94.80	90.01
	Type 2	96.89	99.04	95.98	95.79	96.34	97.07
	Type 3	-	-	67.35	19.91	36.21	78.99

**Table 7:** The Rand Index values for the PSO-KM and the KM algorithms.

	PSO-KM	KM
**RTER**	0.4817	0.4767
**RTIR**	0.4652	0.4600
**LTER**	0.5172	0.5127
**LTIR**	0.4620	0.4587
